# Oxygen Extraction Ratio (OER) as a Measurement of Hemodialysis (HD) Induced Tissue Hypoxia: A Pilot Study

**DOI:** 10.1038/s41598-018-24024-8

**Published:** 2018-04-04

**Authors:** Silverio Rotondi, Lida Tartaglione, Maria Luisa Muci, Alessio Farcomeni, Marzia Pasquali, Sandro Mazzaferro

**Affiliations:** 1 Nephrology and Dialysis Unit, ICOT Hospital, Polo Pontino Sapienza University of Rome, Rome, Italy; 2 Department of public health and infectious diseases. Section of statistics, “Sapienza” University of Rome, Rome, Italy; 3 Nephrology and Dialysis Unit, Policlinico Umberto I, Rome, Italy; 4 Department of Cardiovascular Respiratory Nephrologic Anesthetic and Geriatric Sciences, Sapienza University of Rome, Rome, Italy

## Abstract

HD tissue hypoxia associates with organ dysfunctions. OER, the ratio between SaO_2_ and central-venous-oxygen-saturation, could estimate oxygen requirements during sessions, but no data are available. We evaluated OER behavior in 20 HD patients with permanent central venous catheter (CVC) as vascular access. Pre-HD OER (33.6 ± 1.4%; M ± SE) was higher than normal (range 20–30%). HD sessions increased OER to 39.2 ± 1.5% (M ± SE; p < 0.05) by 30′ and to 47.4 ± 1.5% (M ± SE; p < 0.001) by end of treatment (delta 40%). During HD sessions of the long and short interdialytic intervals, OER values overlapped, suggesting no influence of patient’s hydration status shifts. OER increased (p < 0.05) after 30′ of isolated HD (zero ultrafiltration), but not during isolated ultrafiltration (zero dialysate flow), suggesting a role for blood-membrane-dialysate interaction, independent of volume reduction. In ten patients, individual variability of pre-HD OER was low and repeatable (maximum calculated difference over time 6.6%), and negatively correlated with HD-induced OER increments (r = 0.860; p < 0.005), suggesting a decline in the adaptive response along with resting OER increments. In 30 prevalent patients, adjusted multivariate analysis showed that pre-HD OER (HR = 0.88, CI 0.79–0.99, p = 0.028) and percent HD-induced OER (HR = 1.04, CI 1.01–1.08, p = 0.015) were both associated with mortality, with threshold values respectively <32% and >40%. In HD patients with CVC as vascular access, OER is a cheap, easily measurable and repeatable parameter useful to assess intradialytic hypoxia, and a potential biomarker of HD related stress and morbidity, helpful to recognize patients at increased risk of mortality.

## Introduction

Hemodialysis (HD) sessions imply rapid changes in patients’ blood volume and composition, responsible for the so-called “dialytic stress”, which includes intradialytic events (cramps, headache and hypotension) and post HD fatigue. There is evidence that besides clinical symptoms, HD sessions induce some mild degree of organ ischemia mostly occurring without symptoms, but responsible for transient dysfunction (e.g. myocardial stunning or chronic ischemic brain injury), and, in the long term, for organ damage^1–3^. These subtle intradialytic ischemic episodes are clearly referable to a mismatch between oxygen delivery and oxygen consumption^3^ induced by rapid blood volume reduction^4^ and/or other HD related biochemical changes (e.g. pH shifts or complement activation). Non-HD related factors like low resting *arterial oxygen saturation* (SaO_2_) secondary to pulmonary or cardiovascular diseases or anemia can clearly add to the risk and severity of this phenomenon^4–7^. Currently, it is not possible to reliably assess the occurrence and entity of peripheral oxygen mismatch during HD^4,6^, while, from a pathophysiologic point of view, this could represent a useful parameter to monitor. We hypothesized that Oxygen Extraction Ratio (OER), i.e. the ratio between SaO_2_ and *central venous oxygen saturation* (ScvO_2_), which is a reliable and commonly employed parameter to assess oxygen consumption, could be useful for this purpose. Normal OER averages 20–30% and can increase up to 70% in healthy humans during cycle exercise^8^. In addition, values over 50% are considered indicative, in intensive care unit (ICU) patients, of hemodynamic shock, worse prognosis and lower survival rate^9–11^. Nephrologists, who are less familiar with respiratory parameters of acid base balance, may be reluctant to use OER. Further, ScvO_2_, the second parameter necessary to calculate OER, cannot be measured in every hemodialysis patient, but only in those with central venous catheter (CVC) as vascular access. Actually, to the best of our knowledge, no OER data are available in HD patients and only few papers deal with ScvO_2_, which is less precise than OER.

To verify if OER could represent a way of monitoring tissue response and adaptation to intradialytic stress, we planned the present study to evaluate in detail the relationship between OER and HD. Our aims were the following: to describe the changes, if any, of OER during HD sessions; to evaluate if OER is sensibly affected by the interdialytic changes in patient’s hydration status; to verify if OER is differently induced by isolated UF dialysis (iUF) or isolated Diffusion dialysis (iD); to establish its stability in the individual patient, and to evaluate possible associations with clinical outcomes.

## Materials and Methods

### Study design and subjects

**Figure 1:**
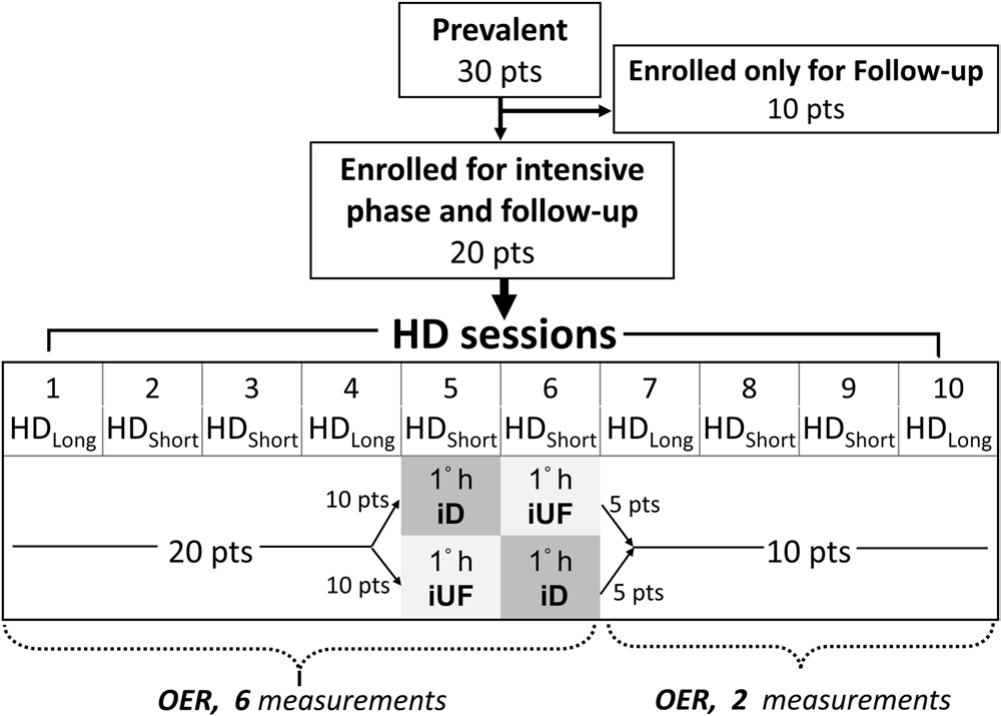
Flow chart of the study. 20 patients were enrolled for the intensive phase of the study which lasted for ten consecutive hemodialysis sessions. In every subject, OER was measured six times during each of the first 6 HD sessions, while in dialysis sessions 7 to 10 OER was measured only twice, in ten patients only. Ten further patients were enrolled for the clinical follow-up phase. HD_Long_ = Hemodialysis following long interdialytic interval; HD_Short_ = Hemodialysis following short interdialytic interval; iD = isolated dialysis; iUF = isolated ultrafiltration.

We planned a prospective, single Center, observational study involving all the HD patients receiving treatment thrice weekly in our Unit at Polo Pontino, Sapienza University of Rome, by means of a CVC. The protocol study, which included an intensive phase with repetitive OER measurements within 3 weeks, and a longer clinical observation period of at least three months, was submitted and approved by the local EC (ASL Rm2; prot. N°107055/2017) and was in accordance with the declaration of Helsinki. Eligible patients gave informed consent. Inclusion criteria were: age ≥18 years, chronic HD treatment since at least three months by means of permanent jugular CVC, no evidence of acute underlying illness. Exclusion criteria from the intensive phase were: presence of arteriovenous fistula, fistula surgery scheduled within 30 days, evidence of displaced or malfunctioning CVC, Chronic Obstructive Pulmonary Disease or SaO_2_ < 90% in resting condition, severe refractory anaemia (Hb < 9 g/dl despite adequate Erythropoietin and iron supplement therapy), congestive heart failure (NYHA class ≥ II) and severe peripheral vascular ischemia. From a total of 30 prevalent cases with CVC, 10 were excluded from the intensive phase (three because of contemporary presence of AVF, three because of AVF placement scheduled within one month, three because of severe peripheral vascular ischemia and one because was going to move to another Unit). The remaining twenty cases entered the intensive phase, and were allocated into small groups to be studied within a maximum of 10 consecutive HD treatments, as illustrated in Fig. 1. In this group, the entity of OER changes during HD and the influence of patient’s hydration status were evaluated during the first 4 HD sessions. For this purpose, we sampled OER six times during each session (OER_6m_ = before HD and after 15′, 30′, 60′, 120′, and end of treatment) in order to obtain two trends after the long interdialytic intervals (sessions 1 and 4, identified as HD_Long_,) and two trends after the short interdialytic intervals (sessions 2 and 3, identified as HD_Short_) (Fig. 1). The relative roles of dialysis and ultrafiltration (UF) were studied during the 5^th^ and 6^th^ HD sessions, again by measuring OER 6 times (OER_6m_). During the first hour of these HD_short_ sessions, either dialysate flow (HD-iUF) or ultrafiltration (HD-iD) were excluded, while standard treatment was resumed in the following hours. To increase homogeneity, in ten patients the sequence was first HD-iUF and then HD-iD, while in ten it was in the reverse order (first HD-iD and then HD-iUF) (Fig. 1). OER values of the iUF and of the iD sessions were then pooled together, independently of the collection order. Further, to evaluate variability and repeatability of OER in the individual patient, we measured it only twice (OER_2m_ = before and at the end of treatment) in the HD sessions from 7 to 10, in ten randomly allocated patients. By pooling these data with values of sessions 1 to 6, a total of ten consecutive pre- and post-HD OER values were obtained in ten patients.

Finally, to explore the possible relationship of OER with clinical outcomes, we considered the whole population of 30 prevalent patients since in all of them we had at least two time-spaced OER measurements (obviously obtained before AVF placement) within a minimum follow-up of 3 months.

Blood for gas analysis was sampled from the arterial line of the dialysis equipment at the established times. To avoid pre-analytical artifacts, handling of blood for gas analysis was standardized according to the manufacturer′s instructions. During all the HD study-sessions, patients wore a finger oximeter for SaO_2_ measurement, whose values were recorded concomitantly to blood samplings.

### Measurements and Instruments

OER was calculated with the following formula:

$${\rm{OER}}=[({{\rm{SaO}}}_{2}-{{\rm{ScvO}}}_{2})/{{\rm{SaO}}}_{2}]\times 100.$$

SaO_2_ was monitored during all the HD study-sessions by means of a peripheral pulse-oximetry device (Max Puls Two, Eurosanitas Italy; accuracy of SaO_2_ measurement = 2%). ScvO_2_ was measured at established times during HD sessions by sampling blood from the arterial line of the dialysis circuit and immediately performing blood gas analysis with a dedicated equipment (GEM 4000 premier, Instrumentation Laboratory Italy; accuracy of SO_2_ measurement = 2%).

### Hemodialysis

All patients received standard bicarbonate dialysis, according to their individual prescriptions of electrolyte concentrations and blood and dialysate flows. Dialyzer membrane was polyaril etheresulphone in all, with surface tailored to the patient body surface. Dialysis machines were all equipped with devices to record the relative changes in blood volume (BV) produced during therapy. The standard policy of our Center is to keep ultrafiltration rate at ≤ 10 ml/kg/h with a dialysate temperature between 35.5 and 36.0 °C.

### Statistical analysis

Data are reported as median (and IQR, 50%) or mean (±SE) as appropriate. Absolute and relative differences of parameters between consecutive visits were computed and described. Statistical significance of various predictors and time was evaluated by mixed-effects linear regression, where a subject-specific random effect was used to capture dependence arising from repeated measures on the same subject. The global level for statistical significance was pre-specified as 5%, where multiple tests were adjusted through Bonferroni correction. In order to evaluate stability/replicability of OER measurements we computed the subject-specific relative differences. As customary, stability was defined as lack of trends and stagionality over time, and maximal estimated subject-specific relative difference below 10%. Association of relevant predictors (pre-HD OER, HD-induced OER) with time-to-event outcomes was evaluated through stratified Kaplan-Meier curves and associated log-rank tests and/or univariate Cox regression models. At multivariate analyses we used Cox regression models, where the final set of predictors was selected by means of forward selection based on Akaike Information Criterion. Predictors of pre-HD OER and HD-related OER changes were assessed by means of linear regression models.

The software used was R version 3.3.3 (R development core team).

The datasets generated and analysed during the current study are available from the corresponding Author on reasonable request.

## Results

Main clinical and biochemical characteristics of the whole prevalent population, of the 20 patients enrolled in the intensive phase, and, by comparison, of the 10 patients excluded from this phase are shown in Table 1. There were 13 males and 17 females, aged 77 ± 2.3 years, receiving substitutive therapy since an average of 3.0 years (range 1–11 years). Three were obese (10%), eleven (36%) had diabetes and eight (26%) had vascular co-morbidities. As evidenced in the table, length of stay on dialysis (3.7 ± 0.7 vs 2.4 ± 0.6 years, M ± SE; p < 0.02) and BMI (25.1 ± 1.2 vs 22.1 ± 0.7, M ± SE; p < 0.03) were the only clinical and biochemical differences between the two sub-populations of 20 and, respectively, 10 patients.

**Table 1:** Clinical and biochemical characteristics of the whole prevalent population, othe the 20 patients enrolled in the intensive phase and, by comparison, of the 10 patients excluded from this phase. Data are M ± SE.

Patients	(n = 30)	(n = 20)	(n = 10)	20 vs 10 p<
Men/Females; n (%)	13 (43)/17 (57)	8 (40)/12 (60)	5 (50)/5(50)	n.s
Age, yr	77 ± 2.3	75 ± 2.9	76 ± 2.9	n.s
Vintage HD, yr	3.0 ± 0.7	3.7 ± 0.7	2,4 ± 0.6	0.02
BMI, Kg/m^2^	24.1 ± 1.3	25.1 ± 1.2	22.1 ± 0,7	0.03
ABI	0.6 ± 0.06	0.7 ± 0.06	0.6 ± 0.05	n.s
Comorbidities; n (%)	15 (50)	12 (60)	3 (30)	n.s (#)
*Obesity* (*BMI* > 30)	*3 (10)*	*2 (10)*	*1 (3)*	
*Diabetes, n* (%)	*11 (36)*	*7 (35)*	*4 (13)*	
Vascular co-morbidities*	*8 (25)*	*5 (25)*	*3 (30)*	
Hb, g/dl	10.0 ± 0.4	10.3 ± 0.3	9.4 ± 0.4	n.s
Calcium, mg/dl	8.4 ± 0.3	8.5 ± 0.1	8.3 ± 0.22	n.s
Phosphate, mg/dl	5.0 ± 0.5	5.2 ± 0.3	4.6 ± 0.6	n.s
PTH, pg/ml	286 ± 30	338 ± 35	272 ± 26	n.s
ALP, UI/L	79 ± 5,1	84 ± 5,9	81 ± 10	n.s
CRP, mg/dl	2.2 ± 0.5	2.1 ± 0.6	1.5 ± 0.6	n.s
ESR, mm	36 ± 4.7	36 ± 4.7	24 ± 14	n.s
Ferritin, mcg/l	345 ± 43	321 ± 46	337 ± 84	n.s
Transferrin, mg/dl	174 ± 12	179 ± 13	148 ± 16	n.s
Albumin, g/dl	3.1 ± 0.1	3.3 ± 0.06	2.9 ± 0.20	n.s
KT/V	1.40 ± 0.06	1.48 ± 0.04	1.37 ± 0.05	n.s
nPCR, g/Kg/day	1.00 ± 0.07	1.03 ± 0.04	0.95 ± 0.03	n.s

^*^Vascular co-morbidities = Peripheral ischemia and acute Coronary Syndrome.

BMI: Body Mass Index; ABI: Ankle Brachial Index; Hb: Haemoglobin; PTH: intact Parathyroid hormone; ALP: Alkaline Phosphate; CRP: C-reactive protein; ESR: Erythrocyte Sedimentation Rate; nPCR: normalized Protein Catabolic Rate. All comparisons are t-test, except for *X*^2^ test (#) applied for comorbidities.

### Effects on OER of standard HD session and of hydration status (study sessions 1 to 4)

**Figure 2:**
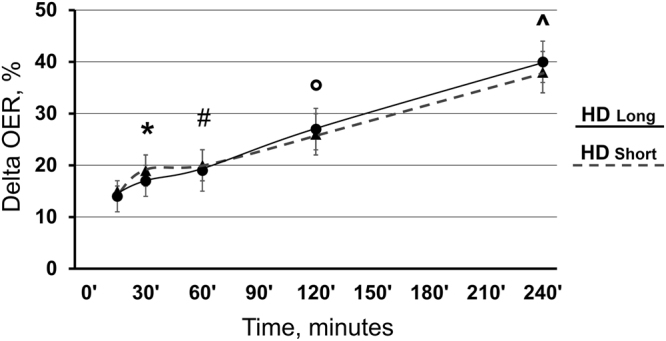
Both HD_Long_ (ANOVA, p < 0.001) and HD_Short_ (ANOVA, p < 0.001) sessions increased OER values. In particular, increments were significant since after 30′ (+17 ± 4% and +19 ± 4% during HD_Long_ and HD_Short_ respectively; M ± SE; p < 0.05 for both), and reached a final rise of +40 ± 4% with HD_Long_ and +38 ± 3% with HD_Short_. No difference was evident between the two sessions. Bonferroni post-hoc test vs basal values: *p < 0.05; ^#^p < 0.005; °p < 0.001; for both sessions.

OER values recorded during the HD_long_ sessions 1 and 4, consistently overlapped and were averaged. Mean pre-HD OER was higher than the normal reference range (33.6 ± 1.4% *vs* a recommended range of 20–30%). During therapy, OER increased by 17 ± 4% (p < 0.05; Fig. 2) since after 30′ and continued to increase progressively up to + 40 ± 4% (p < 0.001; Fig. 2) at the end of treatment. The absolute OER values during each HD_Long_ session are reported in Table 2. SaO_2_ (basal value 97.3 ± 0.3, M ± SEM) was remarkably stable during the whole sessions (values provided in Supplemental Table 1). As expected, ScvO_2_ and BV decreased progressively and significantly (p < 0.001; Supplemental Fig. 1a,b), and no correlation existed between OER (either absolute values or percent change) and BV. In these two HD_long_ sessions, with an average UF rate of 9.7 ml/Kg/h (total UF = 2.2 ± 0.11 L), no clinical symptom occurred and blood pressure and heart rate did not change (Supplemental Table 1).

**Table 2:** OER values (M ± SE) during HD_long_ and HD_short_ sessions in 20 patients.

HD session	0′	15′	30′	60′	120′	240′	Anova
HD_long1_	34.5 ± 1.7	39.3 ± 1.6	40.2 ± 1.8*	41.3 ± 1.4^**#**^	43.4 ± 2.0**°**	47.8 ± 1.4°	<0.001
HD_long2_	33.1 ± 1.4	37.3 ± 1.3	38.2 ± 1.3*	39.3 ± 1.5^**#**^	42.4 ± 1.8**°**	47.0 ± 1.7°	0.001
HD_Short1_	33.8 ± 1.9	40.2 ± 2.7	41.3 ± 1.8*	42.4 ± 1.6^**#**^	42.0 ± 2.1**°**	45.9 ± 2.4°	<0.004
HD_Short2_	36.2 ± 2.0	40.6 ± 2.9	42.2 ± 2.4*	42.0 ± 2.6*	42.9 ± 3.2*	45.9 ± 2.3**°**	0.003

Bonferroni Post-hoc test vs basal values: *p < 0.05; # p < 0.005; °p < 0.001.

Similarly to HD_Long_, OER values consistently overlapped before and during treatment in the HD_short_ sessions 2 and 3, and we averaged them. The OER increment was significant since after 30′ (+19 ± 4%; p < 0.05) and progressively peaked at +38 ± 3% (p < 0.001) by the end of therapy, showing, as illustrated in Fig. 2, an almost perfect overlap with values during HD_Long_. The absolute values of OER during each HD_Short_ session are given in Table 2. SaO_2_ (basal value 97.3 ± 0.2, M ± SEM), showed remarkable stability (Supplemental Table 1). ScvO_2_ and BV progressively decreased (p < 0.001; Supplemental Fig. 1a,b), again without correlation between OER and BV. In these two HD_short_ sessions, Uf averaged 7.7 ml/Kg/h (total Uf = 2.1 ± 0.60 L), without clinical symptoms and without differences in blood pressure and heart rate (Supplemental Table 1).

### Effects on OER of iD and iUF (sessions 5 and 6)

**Figure 3:**
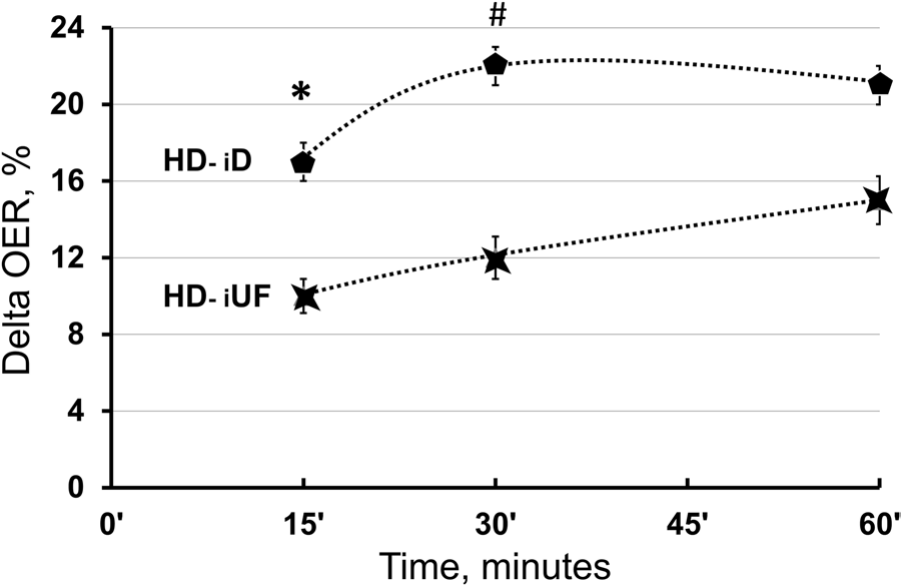
OER increment during iD was greater than during iUF (ANOVA p < 0.04) and was significantly different after 15′ and 30′ but not after 60′. Bonferroni post-hoc test, iD *vs* iUF: *p < 0.05; ^#^p < 0.01.

The relative roles of iD and iUF were evaluated during the first hour of the 5^th^ and 6^th^ HD_short_ sessions, in an alternate sequence involving two groups of ten patients each (Fig. 1). During iD, UF rate was kept to the minimum allowed by the machine, while during iUF, dialysate flow was set to zero. After 15′, 30′ and 60′, OER increased by 17 ± 4, 22 ± 4 and 21 ± 5% during iD and respectively by 10 ± 3, 12 ± 4 and 15 ± 4% during iUF. Compared to basal, these increments were significant during iD (ANOVA, p < 0.05) but not during iUF. Further, during iD the OER increment was greater than during iUF after 15′ (p < 0.05) and 30′ (p < 0.01) but not after 60′ (Fig. 3). The absolute values of OER during iD and iUF sessions are shown in Table 3. During this observation, blood pressure and SaO_2_ were stable (Supplement Table 1), while ScvO_2_ and BV dropped both during iD (p < 0.01 and p < 0.05, respectively, Supplemental Fig. 1c,d), while only BV dropped during iUF (p < 0.01; Supplemental Fig. 1d). The drop of BV with iUF was greater than with iD at 30′ (p < 0.01) and at 60′ (p < 0.05; Supplemental Fig. 1d). When standard HD therapy was resumed, OER values progressively increased became closer, and reached final, comparable, post-HD values of 47 ± 2.0% and 46 ± 1.6%, respectively (Table 3 and Supplemental Fig. 2).

**Table 3:** OER values (M ± SE) during iD and iUF sessions in 20 patients.

HD session	0′	15′	30′	60′	120′	240′	Anova
HD_Short_**, iUF**	33.0 ± 1.4	35.9 ± 1.5	36.6 ± 1.4	37.5 ± 1.5	42.3 ± 1.8°	47.2 ± 2.0°	0.001
HD_Short_**, iD**	32.4 ± 1.4	37.6 ± 1.4	38.8 ± 1.1*	38.4 ± 1.5*	42.3 ± 1.9°	46.4 ± 1.6°	0.001

Bonferroni Post-hoc test vs basal values: *p < 0.05; °p < 0.001.

### OER stability (OER_2m_, study sessions 7 to 10)

**Figure 4:**
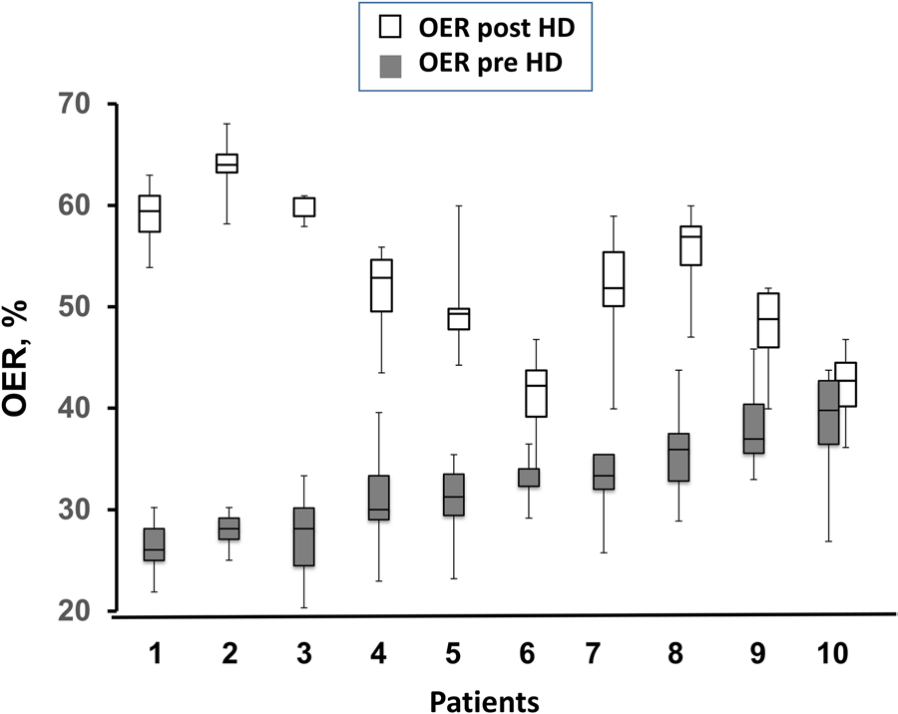
Individual variability (Median, IQ50%, and range) of pre- and post-HD OER in each of the ten patients recorded ten times. Maximal variation over time of pre-HD values was estimated to be 6.6%. Values are shown in increasing order of pre-HD OER.

**Figure 5:**
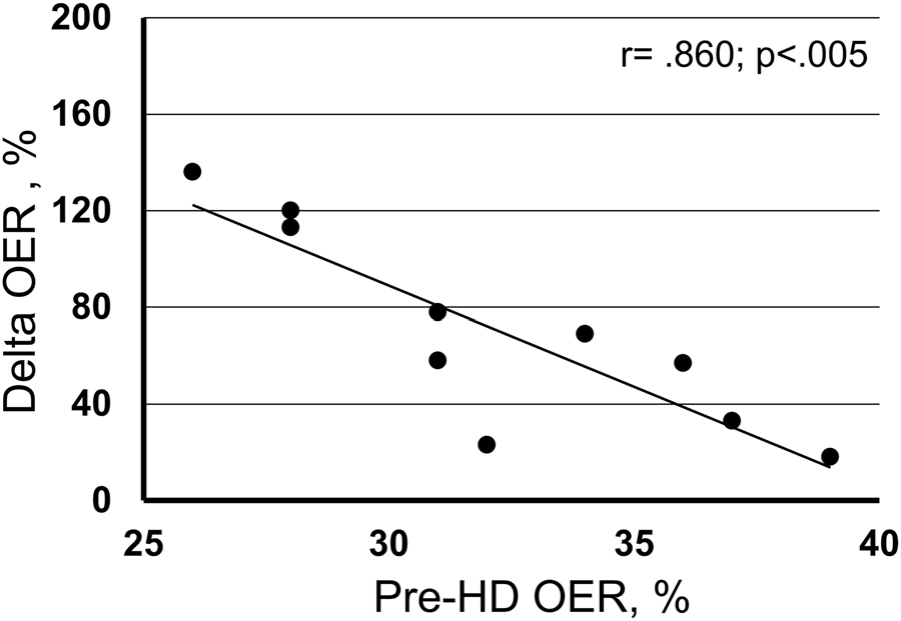
In ten patients, each evaluated ten consecutive times, mean pre-HD OER was strongly and negatively correlated with percent increments.

In ten patients, we measured OER only before and after HD (OER_2m_) in four more sessions and pooled the results with those available in the previous sessions. The individual variability of pre- and post-HD OER (M, IQR and range) observed in these patients is illustrated in Fig. 4, where values are ordered in progressively increasing basal values. The maximum calculated difference over time of baseline OER in the individual patients was 6.6%, pointing to substantial stability and repeatability of the parameter. To be noticed, in this figure post-HD OER did not increase along with the increasing basal values. Indeed, as illustrated in Fig. 5, we found a strong negative relationship between pre-HD OER and OER increments in these ten patients (r = −0.860; p < 0.005). This relationship was confirmed to be negative and significant even in the whole population of twenty patients (r = −0.583; p < 0.01) and, separately, in the other ten patients (r = −0.688; p < 0.02) in whom we had a single value of pre- and post-HD OER.

### OER and mortality

**Figure 6:**
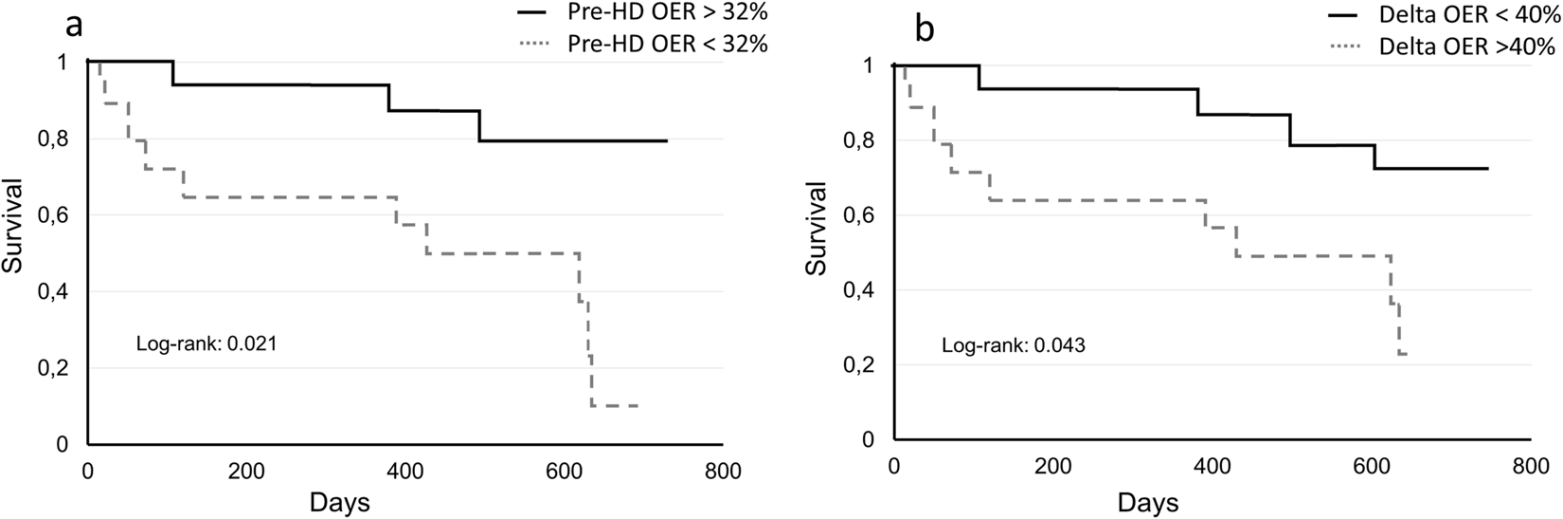
Survival curve according to pre-HD OER (**a**) and HD-related percent OER increments (**b**). Mortality rate was significantly greater in patients with pre-HD OER < 32% and in those with delta OER > 40%.

In the whole population of 30 patients, during a follow-up of 15 ± 1.5 months (range 3–23), we recorded 13 deaths (four infections (13%), three coronary heart disease (10%), three neoplasia (10%) and three protein energy wasting (10%). Pre-HD OER values were negatively associated with all-cause mortality in univariate (HR = 0.9, CI 0.82–0.98, p = 0.015) and multivariate analysis adjusted for age, sex, diabetes and dialysis duration (HR = 0.88, CI 0.79–0.99, p = 0.028). Similarly, associated with mortality in univariate (HR = 1.04, CI 1.01–1.08, p = 0.011) and multivariate adjusted analysis (HR = 1.04, CI 1.01–1.08, p = 0.015), was the percent HD-induced OER increment, this time with a positive relationship. To evaluate if OER distinguished different populations, we considered two groups of patients according to pre-HD OER and HD-related OER changes. As illustrated in Table 4, pre-HD OER < 32% and percent OER increments > 40% were both associated with significantly greater mortality (p < 0.01 for both). These results were confirmed in the corresponding survival curves (Kaplan-Meier log rank test = 0.021 and 0.043 respectively for pre-HD and percent OER increments) (Fig. 6a,b).

**Tabelle 4:** Clinical and biochemical characteristics of population divided according to pre-HD and delta OER value. Data are M ± SE.

Patients, (n.)	Pre-HD OER		p<	Delta OER		p<
	OER ≤ 32% (15)	OER > 32% (15)		Delta OER ≥ 40% (14)	Delta OER ≤ 40% (16)	
Men/Females; n (%)	8 (53)/7 (47)	5 (33)/10 (66)		7 (50)/7(50)	6 (37)/10 (63)	
Age, yr	76 ± 3.0	73 ± 3.4	n.s	75 ± 3	76 ± 3	n.s
Vintage HD, yr	3.0 ± 0.7	4.0 ± 0.6	n.s	3.0 ± 0.7	3.5 ± 0.6	n.s
Comorbidities; n (%)	7 (46)	8 (53)	n.s	6 (42)	9 (68)	n.s
*- Obesity* (*BMI* > 30)	*0*	*3 (20)*		*1 (7)*	*2 (12)*	
*-Diabetes, n* (%)	*7 (46)*	*4 (26)*		*7 (50)*	*4 (25)*	
- Vascular co-morbidities*	*3 (20)*	*5 (33)*		*4 (28)*	*4 (25)*	
Follow-up, months, (*range*)	12 ± 2.2 (23–3)	19 ± 1.1 (23–3)	n.s	14 ± 1,6 (*21–3*)	16 ± 2 (*23–3*)	n.s
All-cause mortality, n (%)	10 (67)	3 (20)	0.01	9 (64)	4 (25)	0.01
*- Infection*	*3 (20)*	*1 (7)*		*3 (33)*	*1 (25)*	
*- Acute coronary syndrome*	*3 (20)*	*0*		*2 (22)*	*1 (25)*	
*- Neoplasia*	*2 (13)*	*1 (7)*		*2 (22)*	*1 (25)*	
- *Protein energy wasting*	*2 (13)*	*1 (7)*		*2 (22)*	*1 (25)*	
OER first measurement, %	27 ± 0.8	37 ± 1.3	0.001	29 ± 1	36 ± 2	0.001
Delta OER, %	48 ± 5.2	33 ± 4.7	0.04	59 ± 2	24 ± 2	0.001
OER LOCF^#^, %	34 ± 2.2**	38 ± 1.7	n.s	37 ± 2.4**	35.0 ± 1.6	n.s

^(^*^)^Vascular co-morbidities = Peripheral ischemia and acute Coronary Syndrome§; ^(#)^LOCF: last observation carried forward.

All comparisons are t-test, except for *X*^2^ test (#) applied for comorbidities and mortality.

^(^**^)^p < 0.01 vs OER first measurement.

## Discussion

In our study, we demonstrate that OER changes significantly during HD sessions, is not affected by the interdialytic shifts of patient’s hydration status, is differently induced by iD and iUF and is stable and repeatable in the individual patient. Further, we found a significant association of both pre-HD OER and percent increments of OER with all-cause mortality. Therefore, resting OER and OER increments can be considered potentially useful biomarkers of the oxygen needs in uremia and of the HD-related hypoxic stress, possibly helpful to stratify mortality risk in the individual patient.

Our data indicate that, by means of OER progression during sessions, we can easily quantify the commonly appreciated intradialytic increment in oxygen need^4,12^. Further, the evidence that the average shifts in hydration status occurring within dialysis treatments do not affect the process of oxygen extraction, suggests that we could obtain the resulting clinical information in any weekly session, at least in experimental conditions like ours (i.e. with moderate UF rate and in the absence of intradialytic symptoms). The evidence in our study that dialysis procedure per se, even without UF, plays a significant role in the increment of OER is original. In fact, in our observation OER increased significantly since after 30′ of iD and, remarkably, in higher amounts than it did with iUF. This iD-related quick increment indicates that, in the absence of UF, factors like biocompatibility^13^, pH^14^ and electrolyte^15^ shifts, increase the need of oxygen at tissue level, more than iUF does. Indeed, the recorded increment in oxygen extraction with iUF was slower, pointing to a reduced or smoother hypoxic stress, and this finding is in agreement with the common observation of improved hemodynamic stability during iUF dialysis.

Importantly, the temporal changes of OER in our study, within 30 minutes of HD, correspond with the reduction of microvascular perfusion and of parenchymal myocardial function evidenced in HD patients only by means of Magnetic Resonance Imaging (MRI), and not by standard parameters (like blood pressure and heart rate)^1,16^. Similarly, a PET scan after 30′ of iD without UF evidenced a significant reduction of regional myocardial blood flow^17^. Since it is possible that an increased oxygen consumption would precede or associate with episodes of tissue ischemia, we can hypothesize that OER could unravel patients suffering or at increased risk of dialysis induced tissue hypoxia. The quick increment of OER described in our study becomes thus a plausible biomarker of dialytic stress, helpful to find out, in the individual patient, the possibly involved factor among those commonly claimed to explain dialysis related morbidity. In our opinion, the evidence produced by MRI and PET that the ischemic burden of HD occurs early, reinforces the diagnostic potential of OER as a marker of hypoxia.

Another original and important finding in our study is that pre-HD OER is sufficiently stable and repeatable in the single patient. This suggests that OER could be used as a routine tool of clinical stability in our fragile patients. Importantly and unexpectedly, pre-HD OER levels in our patients were higher than the commonly accepted normal reference (i.e. 20–30%) and reached post-HD values that, in ICU, are considered diagnostic of hemodynamic shock. Since none of our patient was symptomatic or suffered congestive heart failure or COPD and all of them had adequate peripheral oxygenation (evidenced by normal SaO_2_), this must be related to reduced ScvO_2_ even before starting treatment. Indeed, resting ScvO_2_ in our patients averaged 67 ± 1.7.% which is lower than the 70% threshold recommended (together with low Hb) by the American Heart Association to decide blood transfusion in critically ill children^18^. Recently, in 232 HD patients with CVC as vascular access, Chan *et al*.^19^ evaluated retrospectively the association between ScvO_2_ and mortality. With an observation period of 3 years, they found better survival in those patients in the upper tertiles of ScvO_2_. Given the strict relationship between ScvO_2_ and OER, our hypothesis that OER could be a useful diagnostic tool in HD is confirmed. Conceptually, as the same Authors of this paper underline, OER is more precise and reliable than ScvO_2_ since it also includes the measurement of peripheral arterial oxygenation (SaO_2_). In addition, in our study patients with higher pre-HD OER values had lower increments at the end of treatment, while those with lower pre-HD OER values (within normal range) were apparently able to increase definitely better the extraction of oxygen from peripheral blood, as predictable in normal subjects^8^. Alternatively, the latter group seems to face a significantly higher hypoxic burden. While the clinical relevance of this inverse relationship deserves better appreciation, it clearly indicates that the adaptive response to hypoxia during HD can be quite different among patients and that OER could be helpful to distinguish them. Hypothetically, the OER response could characterize those at increased risk of asymptomatic, intra-HD ischemia. As a comparison, in non-renal patients, OER has been recently reported to be useful to recognize among anemic children receiving cardiac surgery those who would benefit most from blood transfusions^20^, and to be a major determinant of exercise capacity in adult patients with heart failure and preserved ejection fraction^21^. These clinical observations further underline the potential usefulness of OER.

Importantly, in our study OER values associated with mortality. The negative association of pre-HD OER (i.e., lower values = higher mortality) are at variance with the abovementioned data by Chan *et al*.^19^ showing better survival of patients in the higher S_C__V_O_2_ tertile (then with presumably lower OER). However, the population in that study is definitely younger than our and does not show the substantial reduction of ScvO_2_ at the end of dialysis, invariably described in the literature^4,7^. In any case, since uremia is generally regarded as a clinical condition of increased oxygen demands^7^, an adaptive response which increases OER even in resting condition seems plausible and in accordance with the general finding of invariably low pre-HD ScvO_2_ values in HD patients^4,12,19^. Therefore, having lower values could also indicate absent or insufficient adaptation to uremia. As for the positive association of delta OER with mortality, it seems much easier to explain since it is in agreement with the hypothesis of increased hypoxic stress during dialysis and, eventually, with the finding of increased morbidity and mortality. All in all, our data suggest that adapted patients (those with increased pre-HD OER) suffer less the HD-related hypoxic stress (lower OER increments) as compared to not-adapted (or maladapted/not-still-adapted) patients.

A practical final consideration to do is that OER measurement does not imply cost increments, is easily accessible to most if not all of the dialysis units, and is conceptually sound to appreciate the recently revealed importance of intra-dialysis tissue hypoxia.

Our study has limitations. The number of patients included in the follow-up data is rather low to firmly establish if OER associates with dialysis-related hard-outcomes. However, our pilot study can be regarded as essential to unravel the behavior of a parameter never evaluated before in HD patients. As such, it is preliminary to any large prospective clinical study. Another limitation is that we did not measure the time and rate of OER recovery in the hours following HD treatment. While such an observation would have made the study more complete and could have ruled out if, by chance, the OER recovery-time overlaps with the so called “post-HD fatigue” thus reinforcing its role as a biomarker, it is certainly not essential for our conclusions. Finally, since OER can be measured only in patients with CVC who may suffer from increased cardiovascular complications, it must be regarded as limited to this type of patients and then of reduced practical application. However, while the prevalence of patients with permanent CVC is not marginal, the clinical information obtainable with OER in these patients could be translated to those with a fistula as vascular access. Further, recent researches suggest that peripheral venous O_2_ saturation could be measured non-invasively thus allowing to estimate peripheral oxygen extraction in patients without central vein catheterization^22^.

Strengths of our study include the prospective design and the originality of the observation, which allowed to characterize, for the first time in HD patients, the performance of OER.

We conclude that in HD patients with CVC as vascular access, OER is an easy, cheap, measurable and repeatable parameter that reflects the intra-HD related parenchymal hypoxic stress. The consistency of the results with available clinical studies on intradialytic ischemia and the number of potential clinical applications of OER suggested by our results, claim for further observations and developments for this parameter.

## Electronic supplementary material

Supplemental material
