# Supplementary material for: Oxygen Extraction Ratio (OER) as a Measurement of Hemodialysis (HD) Induced Tissue Hypoxia: A Pilot Study
Source: Sci Rep. 2018 Apr 4;8:5655. doi: 10.1038/s41598-018-24024-8 (PMC5884820; doi:10.1038/s41598-018-24024-8)
Supplement: Supplementary file 1 — Supplemental material [file 41598_2018_24024_MOESM1_ESM.pdf]

Supplemental Material

Oxygen Extraction Ratio (OER) as a Measurement of Hemodialysis (HD) Induced Tissue Hypoxia. A Pilot Study

Silverio Rotondi[1], Lida Tartaglione[1], Maria Luisa Muci[1], Alessio Farcomeni[2], Marzia Pasquali[3] and * Sandro Mazzaferro[1,4]

[1] Nephrology and Dialysis Unit, ICOT Hospital, Polo Pontino Sapienza University of Rome.

[2] Department of public health and infectious diseases. Section of statistics. “Sapienza” University of Rome, Italy

[3] Nephrology and Dialysis Unit, Policlinico Umberto I, Rome. Italy

Department of Cardiovascular Respiratory Nephrologic Anesthetic and Geriatric Sciences, Sapienza University of Rome. Italy

Corresponding author: Prof. * Sandro Mazzaferro, “Sapienza” University of Rome

Address: Viale del Policlinico 155 00161, Rome

Tel: 0039 0773 6513097

Fax: 0039 0773 6513254

E-mail: * sandro.mazzaferro@uniroma1.it

Supplemental table 1. SBP, DBP and SaO_2 (M±SE) during all HD sessions

	Time, min	0’	15’	30’	60’	120’	240’	p
HD-long	S.B.P., mmHg	121±4	122±5	120±3	118±3	115±4	122±5	n.s
	D.B.P., mmHg	65±3	64±2	65±2	65±2	65±2	66±2	n.s
	SaO_2, %	97.3±0.3	97.3±0.4	97.1±0.3	97.3±0.3	97.3±0.3	93.3±0.3	n.s
HD-short	S.B.P., mmHg	119±4	119±4	118±4	118±2	115±3	120±4	n.s
	D.B.P., mmHg	64±2	64±2	65±2	65±2	65±2	67±3	n.s
	SaO_2, %	97.3±0.2	97.2±0.3	97.2±0.3	97.2±0.3	97.6±0.3	97.6±0.3	n.s
HD-iD	S.B.P., mmHg	122±5	121±5	119±4	120±4			n.s
	D.B.P., mmHg	64±2	64±2	65±2	64±3			n.s
	SaO_2, %	97.0±0.2	97.0±0.3	96.6±0.3	97.1±0.3			n.s
HD-iUF	S.B.P., mmHg	121±4	119±4	120±5	116±4			n.s
	D.B.P., mmHg	63±2	64±2	63±2	64±2			n.s
	SaO_2, %	97.4±0.2	97.0±0.4	96.9±0.3	97.1±0.3			n.s

ANOVA; SBP: systolic blood pressure; DBP: diastolic blood pressure; SaO_2: arterial oxygen saturation;

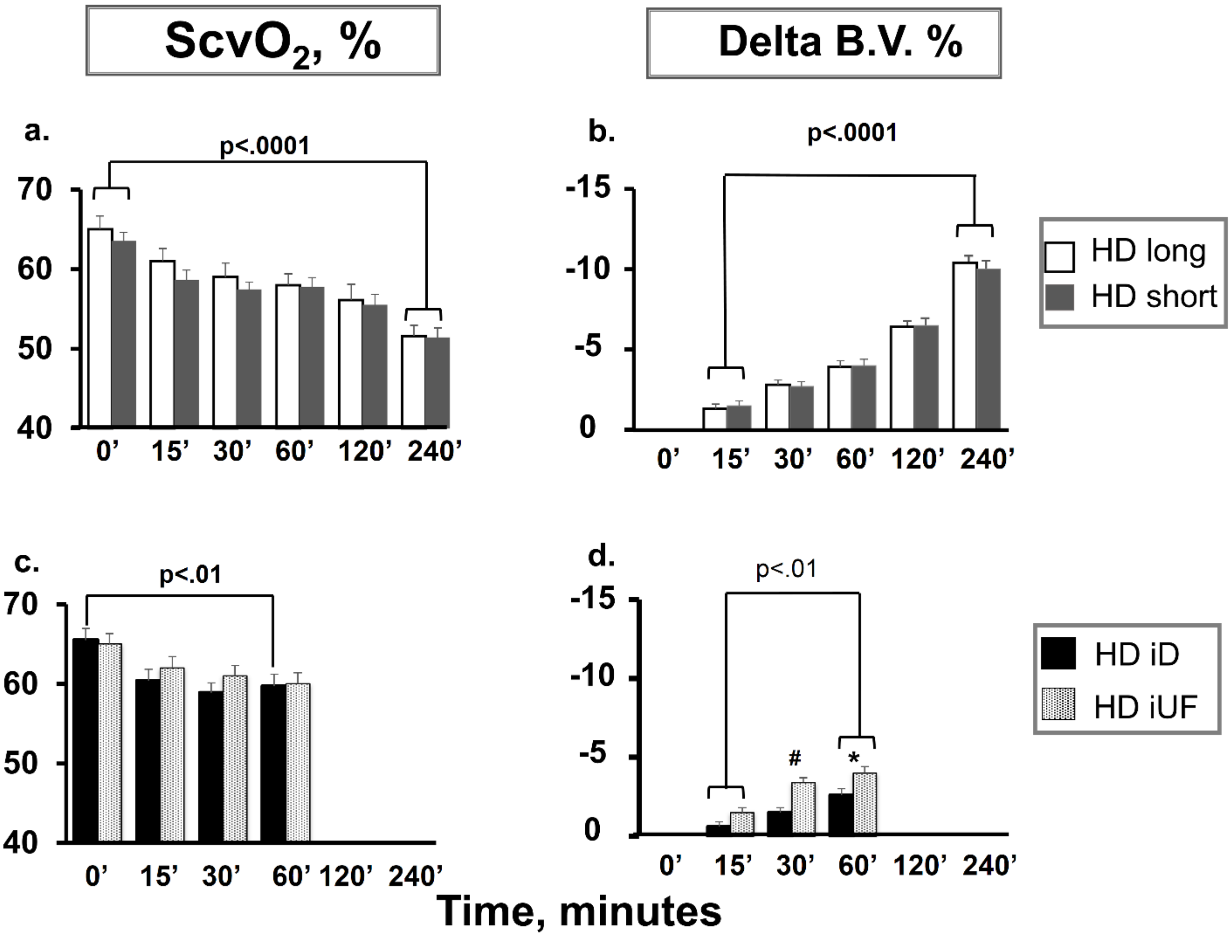

Supplemental Figure 1. Comparison between HDLong and HDShort and between iD and iUF of ScvO2 and BV changes during sessions. a. and b. = ScvO2 and BV changed significantly (Anova, p<0.001) during HDLong and HDShort, but without differences between them. c. = ScvO2 dropped significantly during 60’ of iD (Anova p<0.01) but not during iUF. However, non difference was evident between the two methods; d. = BV changed significantly with both iD (anova, p<0.01) and iUF (p<0.01), and the drop was significantly greater with iUF after 30’ (p<0.01) and 60’ (p<0.05). Bonferroni post-hoc test, iD vs iUF: *p<0.05; # p<0.01.

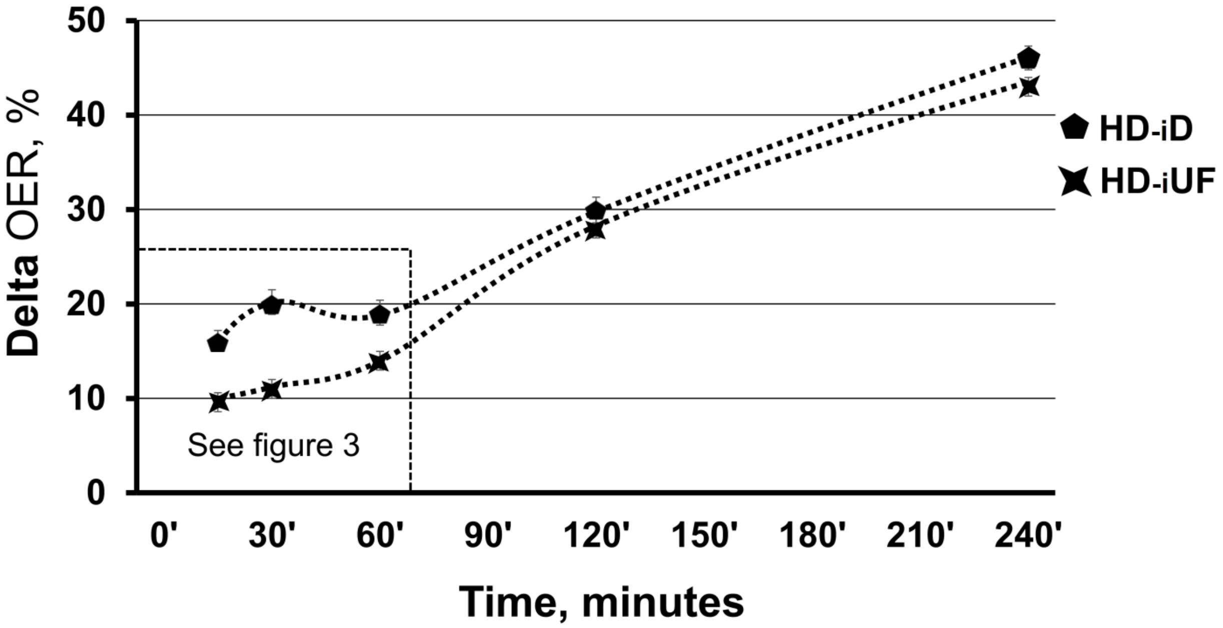

Supplemental Figure 2. After 1h of iD and iUF, standard HD treatment was resumed and resulted in a gradual, progressive approaching of the two OER curves, which reached final comparable values (44±2 and 42±2% respectively).
